# Attitudes of Israeli medical students towards the medical treatment of uninsured migrants

**DOI:** 10.1186/s12909-020-1973-4

**Published:** 2020-03-14

**Authors:** Zohar Mor, Adam Cadesky, Ran Halleluyan, Rivka Sheffer

**Affiliations:** 1 Tel Aviv Department of Health, Tel Aviv, Israel; 2 School of Health Sciences, Ashkelon Academic College, Ashkelon, Israel; 3 Faculty of Medicine, Tel Aviv University, Tel Aviv, Israel

**Keywords:** Curriculum, Medical education, Medical school, Migrant, Minority

## Abstract

**Background:**

Undocumented migration to developed countries poses practical concerns, as migrants are not medically insured. This cross-sectional study aims to appraise the attitudes of Israeli medical students towards the uninsured migrant population.

**Methods:**

Participants from five medical schools in Israel completed anonymous questionnaires in Hebrew, based on the “Medical Students’ Attitudes Toward the Underserved” (MSATU), which assessed students’ attitudes regarding the professional responsibility and societal expectations towards the migrants. It also evaluated students’ views of the migrants as eligible for expensive medical procedures.

**Results:**

A total of 891 students completed the survey with a median age of 28 years. The majority were Jews (*N* = 816, 91.6%) and singles (*N* = 681, 68.5%). Participants in the pre-clinical years were likely to be female and unmarried compared to those in clinical training. They also demonstrated higher scores on professional responsibilities and societal expectations than students in clinical training, but no significant differences were found in their views on expensive medical services. Students of minorities (non-Jews and migrants) scored higher on professional responsibilities and societal expectations.

The scores for professional responsibilities and societal expectations decreased as students progressed in their medical training (Spearman coefficient *p* = 0.04 and *p* = 0.01, respectively). This trend was more apparent in males rather than females.

**Conclusion:**

MSATU scores declined as students progressed through medical school, with females maintaining more favorable attitudes than males. Medical schools should attempt to maintain the enthusiasm and idealism that students possess as they enter medical training and provide clinical experience with migrant populations that allows for cross-cultural communication.

## Background

The number of international migrants worldwide has continued to grow in recent years, reaching 258 million in 2017, with high income countries accepting 64% of all the migrants [1]. Undocumented migration poses both practical and ethical dilemmas for the healthcare systems in many developed countries because undocumented migrants are usually excluded from basic and preventative medical services in countries with universal healthcare. As a result, considerable care is spent in the emergency rooms and hospital departments, which are expected to provide emergency services to all [2]. The economic burden of their medical care is thus shifted onto the tax-paying population. Migration also poses an essential ethical issue regarding the extent of the responsibility of the medical system in the hosting countries, as reflected in social debates in Israel [3].

Israel was ranked 36th in the global Gross National Income per capita index in 2016 with per capita income of $36,100 [4] among its population of approximately 8.2 million citizens. Israel’s population is comprised of 74.5% Jews and 20.8% Arabs, most of which are Muslims [5]. Israel also receives Jewish immigrants, and it is estimated that 25% of the Jewish population were born elsewhere [6].

It is estimated that there are over 200,000 labor migrants living in Israel, of which about 50% are undocumented [7]. Since 2006, more than 50,000 migrants have emigrated from the horn of Africa. Unlike Israeli citizens, who are entitled to full medical health services under the National Insurance Law [8], undocumented migrants are excluded from this act. Only a few services are currently available to address the medical needs of these undocumented migrants. Most of these medical services are available in Tel Aviv, reflecting the greater population of undocumented migrants, and includes two walk-in general health centers, a community clinic for sexually transmitted infections, a tuberculosis dispensary, and mother-and-child health care centers [9].

The attitudes, beliefs and biases that a medical student brings to medical school or may develop during their medical education can shape their approach to medicine and ultimately influence the manner in which they provide care for the migrant population. Migrant health is not included as a subject area in medical schools in Israel, even though students are confronted with the challenges of providing medical care for the migrants during their medical education. Students should develop cross-cultural sensitivity and foster a patient-centered approach to undocumented migrants. Medical students should also be aware that the migrant population often faces communication difficulties due to linguistic and cultural barriers, which hampers their ability to obtain a proper history or to convey medical recommendations. This is exacerbated by the ambiguous coverage that migrants receive in the media and by negative opinions expressed by the Israeli public, which may lead students and healthcare workers to develop negative attitudes toward this patient population, thus affecting their care [10].

Since medical students will interact with migrant patients after they graduate from medical school, it is important to identify negative attitudes before students enter professional practice. This study aims to assess the knowledge and attitudes of Israeli medical students toward the uninsured migrant patient population in Israel. The findings of this study may assist faculty members in shaping medical school curricula by increasing cultural awareness of future doctors and achieving positive attitudes towards individuals who are not medically insured.

## Methods

Students for this cross-sectional study were recruited from all five medical schools in Israel via the student bureaus and secretariats. Each medical student received an electronic message with a link to the survey. In order to increase participation, medical books and otoscopes were raffled off as prizes. Medical education in Israel has two trajectories: the first is a 6-year curriculum, divided into 3 years of pre-clinical studies and 3 year of clinical training. The second is a 4-year program, which is designed for students who already have a bachelor degree, and includes gradual exposure to clinical training. All students are required to complete 1 year of rotating internship after graduation. In 2017, there were 3839 students in both academic programs, and the female to male ratio was 1:1.2. Of those, 1706 (44.5%) were in pre-clinical studies and 2133 (55.5%) in clinical training [11]. For the purpose of this study, we included all students in the 4-years program in the clinical training group, by the relevant year of medical education.

Participants were requested to complete an anonymous questionnaire in Hebrew, which was based on the “Medical Students’ Attitudes Toward the Underserved” (MSATU) [12, 13]. For the purpose of this study, the MSATU has been edited to accommodate the Israeli medical student population by translating from English to Hebrew and adopting the terminology, mainly from the original expression “underserved” to “undocumented migrants”, thus allowing Israeli participants to refer more specifically to the local migrant population (Appendix 1). The questionnaire specifically reminded the students that undocumented migrants are not medically insured.

The MSATU questionnaire contains two subscales that use a 5-point Likert scale, indicating the extent of agreement or disagreement with designated statements (5 = strongly agree, 1 = strongly disagree). The 23 items within the first subscale assess attitudes regarding professional responsibilities and personal or societal roles. The 14 items within the second subscale assess the level in which the migrants can use low cost or expensive medical procedures and services. The responses in the different sub-scales for each individual student were combined to create a total attitude score. Negatively stated items were reverse coded; higher scores indicate more favorable attitudes.

Scores were standardized to T-scores to provide a basis for comparing the magnitude of the effects. For this study, T-scores have a mean of 50 and a standard deviation of 10 (a T-score of 43 is therefore 0.7 of a standard deviation below the mean), as calculated elsewhere [14]. Furthermore, internal consistency was calculated by Cronbach’s alpha for each set of questions. Comparisons between pre-clinical and clinical year students were performed using the Students’ *t*-test for continuous variables and the chi-square test for categorical variables. A spearman coefficient was used to test the association between the school class and the continuous variables of the students’ attitudes. Trend analysis was performed by the chi-square test to yield a linear by linear association between the year in the medical school with professional responsibilities’ and social expectations’ T-scores. A *p*-value < 0.05 was considered statistically significant.

## Results

A total of 891 students completed the survey; 323 (36.3%) were in their pre-clinical years and 568 (63.7%) were in clinical training. Of all participants, 706 (79.2%) were in the 6-year program and 185 (20.8%) in the 4-year program. Participants’ median age was 28 years (25–75% interquartile range: 25–30), and the majority were females (*N* = 461, 51.7%), Jews (*N* = 816, 91.6%) and singles (*N* = 681, 68.5%). Cronbach’s alpha for professional responsibilities and societal expectations, and opinion about basic and expensive services, were 0.87, 0.80, 0.85 and 0.89, respectively.

The standard deviation of the total T-score was 4.6 (range 33.1–61.9). The standard deviation and the ranges of the total T-scores of professional responsibilities, societal expectations, basic services and expensive procedures were 6.9 (range 29.1–65.4), 5.9 (range: 21.9–60.3), 7.3 (range: 8.4–59.1) and 8.6 (range: 24.9–60.9), respectively. The students in pre-clinical years were more likely to be female and unmarried compared to those in clinical training (Table 1). Pre-clinical students demonstrated higher scores on professional responsibilities and societal expectations than students in clinical training, but no significant differences were found in their views on basic or expensive medical services. Minorities showed a different pattern: Non-Jews scored higher on professional responsibilities and societal expectations than Jews (Table 2), and Jewish medical students who were migrants themselves had higher scores on societal expectations than Israeli-born Jews (Table 3). Female students demonstrated higher scores than males on their views of professional responsibilities, societal expectations, and regarding the eligibility of undocumented migrants to access low cost medical services (Table 4).

**Table 1:** Comparison between medical students who participated in the study by stage of studying

	Pre-clinical *N* = 323	Clinical *N* = 568	P
Male	133 (41.2)	297 (52.1)	0.02
Israeli born	286 (88.5)	478 (84.2)	0.07
Jew	299 (92.0)	519 (91.4)	0.8
Married	55 (17.0)	226 (39.8)	< 0.001
Age	25.8 ± 3.6	28.6 ± 2.9	< 0.001
Professional responsibilities^a^	50.1 ± 6.7	49.4 ± 6.9	< 0.001
Societal expectations ^a^	50.5 ± 5.5	49.7 ± 5.5	0.04
Basic services^a^	50.1 ± 6.9	49.9 ± 7.5	0.9
Expensive procedures^a^	49.7 ± 8.7	50.1 ± 8.6	0.6

^a^MSATU T-scores

**Table 2:** Comparison between medical students who participated in the study by religion

	Jew N = 816	Non-Jew *N* = 75	P
Male	391 (47.9)	39 (52.0)	0.5
Married	273 (33.5)	8 (10.7)	< 0.001
Age	27.8 ± 3.4	25.5 ± 3.4	< 0.001
Professional responsibilities^a^	49.6 ± 6.8	54.8 ± 6.4	< 0.001
Societal expectations ^a^	49.8 ± 5.9	51.8 ± 5.0	0.02
Basic services^a^	50.1 ± 7.3	49.2 ± 7.3	0.3
Expensive procedures^a^	50.0 ± 8.6	49.5 ± 8.7	0.6

^a^MSATU T-scores

**Table 3:** Comparison between Jewish medical students who participated in the study by country of birth

	Jew Israeli born *N* = 706	Jew non-Israeli born *N* = 110	P
Male	335 (47.5)	56 (50.9)	0.5
Married	225 (31.9)	48 (43.6)	0.01
Age	27.6 ± 3.5	28.5 ± 2.7	0.02
Professional responsibilities^a^	48.8 ± 7.1	49.7 ± 6.7	0.3
Societal expectations ^a^	48.7 ± 6.4	50.0 ± 5.8	0.04
Basic services^a^	50.1 ± 7.1	49.6 ± 8.3	0.5
Expensive procedures^a^	50.1 ± 8.6	49.8 ± 8.6	0.8

^a^MSATU T-scores

**Table 4:** Comparison between male and female students who participated in the study by gender

	Male *N* = 430 (%)	Female *N* = 461 (%)	
Israeli born	366 (85.1)	398 (86.3)	0.6
Jew	391 (90.9)	425 (92.2)	0.5
Married	149 (34.7)	132 (28.6)	0.06
Age	28.3 ± 3.5	26.9 ± 3.3	0.01
Professional responsibilities^a^	48.9 ± 7.0	51.1 ± 6.7	< 0.01
Social expectations^a^	49.7 ± 6.3	51.3 ± 5.4	0.02
Basic services^a^	49.8 ± 7.3	51.1 ± 7.3	0.03
Expensive services^a^	50.3 ± 8.5	49.7 ± 8.7	0.3

^a^MSATU T-scores

**Fig. 1:**
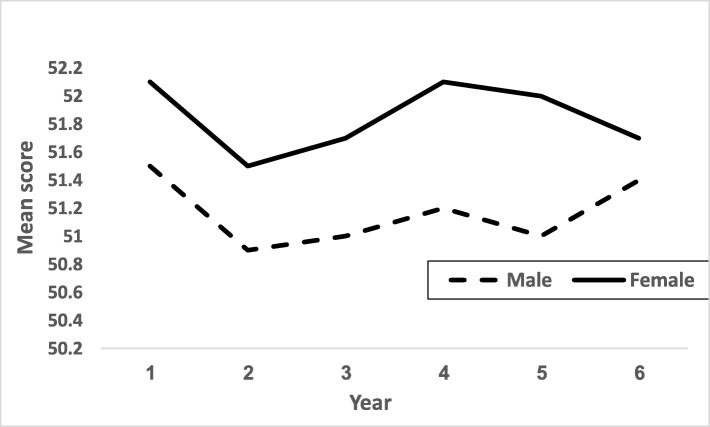
Mean MSATU total scale scores by year of medical school and gender

MSATU scores demonstrated a sinusoidal pattern as the students progressed with each year in medical school: it was initially higher at the beginning of pre-clinical education and then decreased, until it rose again upon entry to clinical training in 4th year, and then gradually decreased again towards graduation (Fig. 1). Female and male students showed similar trends, yet female students scored consistently more favorably than males throughout medical school, *p* = 0.02 for trend.

As medical students progressed through medical school, their views on professional responsibilities and societal expectations generally declined (Table 5). The Spearman coefficient showed an opposite association between the year of medical school and the scores on professional attitudes (r_s_ = − 0.57, *p* = 0.04), which was more predominant in males rather than females (r_s_ = − 0.056, *p* = 0.01 and r_s_ = − 0.36, *p* = 0.04, respectively). The number of years in medical school was also negatively associated with students’ scores on societal expectations (r_s_ = − 0.1, *p* < 0.01), which was also more predominant in males than females (r_s_ = − 0.115, *p* = 0.008 and r_s_ = − 0.89, *p* = 0.03, respectively).

**Table 5:** Mean T-scores for professional responsibility and social expectation by year of medical school

Year	Professional responsibility	Social expectations
1	51.5	50.5
2	51.1	50.3
3	51.2	49.9
4	49.8	49.8
5	49.2	49.9
6	49.1	49.7

## Discussion

Pre-clinical students had more favorable attitudes on professional responsibilities and societal expectations than those already in clinical training. The MSATU scores of both male and female medical students declined in their overall attitudes as they progressed in their medical education, with females demonstrating more favorable scores than males.

The attitudes of medical students towards migrants was characterized by two periods of decline. The first was after entry into medical school and the second was after entry into clinical training. Students’ attitudes evolve during their medical training. They learn to adjust their initial idealism to the reality of financial constraints within the system. They are able to better understand the constraints within the medical system to include non-paying patients, who are not part of the core population of the country. Crandall et al suggested that medical students become more realistic throughout their medical training, and possibly more cynical [15]. The idealism and enthusiasm that students exhibit when starting medical school are later replaced by disenchantment, which coincides with their academic workload and other educational duties, such as final exams [16]. This transformation and decline in ideology during the medical school experience is also impacted when students realize that medical studies are difficult and possibly not as glamorous as portrayed on the media [14].

Female students were shown to be significantly more willing than male students to support the provision of health care services despite an individual’s inability to pay, and to feel a greater sense of personal responsibility, regardless of their year of medical school. This is also corroborated by other studies [17–19]. These studies have shown that female clinicians develop better communication skills which are valuable in building trust between disfranchised populations and the medical provider and that they also tended to engage in a more patient-centered communication than males [20]. Female doctors are also more open to addressing the cultural and linguistic aspects of health care with migrant patients [21].

Minority students who were either non-Jews or non-Israeli born Jews had more positive views regarding societal expectations and professional responsibilities. This finding may reflect their own perception of vulnerability and social instability or their previous experience with different governmental authorities, which increased their sensitivity towards disenfranchised populations [22]. In contrast, medical students who do not belong to minority groups or those who have limited contact with migrants from privileged backgrounds may not fully understand the needs of the vulnerable population [23].

Educators within medical schools train students to develop social sensitivity along with medical knowledge. Students are therefore expected to be able to address the needs of underserved and vulnerable populations. These clinical and communication skills can be developed by using OSCEs (objective structured clinical examination) which involve standardized patients and simulations [24]. In order to improve the professionalism and interpersonal skills of medical students, they should also get an opportunity to work or volunteer in migrant clinics so as to cultivate their social obligation and establish a compassionate medical practice [25]. This approach can be encouraged by the medical schools through positive rewards such as higher academic grades or grants. It has been previously published that medical students and doctors who received cultural competence training had a greater interest in caring for immigrant patients [15] and fostering a more humanistic perspective in their medical care [21]. The International Federation of Medical Students’ Associations (IFMSA) also recommended that migrant issues should be included in students’ core medical curricula, reinforcing the belief that their clinical decisions should be guided by medical needs rather than the legal status of the patients [26]. Having training with a doctor who has experience with migrant health may also influence the students to counterbalance their negative attitudes towards migrant and other underserved populations [27]. Lastly, having medical students interview migrant patients about their personal experience with the health-care system can expose the students to these cultural barriers to optimal healthcare and can foster more ethical attitudes towards care and social responsibility [14, 28].

The study is subject to several limitations. First, the average total response rate was ~ 25% of all the medical students in Israel. However, the percentage of the participants who completed the survey out of all students in each of the schools and each level (pre-clinical or clinical) was not statistically different, which may limit a selection bias (Appendix 2). Second, the students were recruited only from medical schools in Israel. Nevertheless, the MSATU questionnaire is a valid instrument allowing comparisons between students from different countries. Third, responses may be subjected to information bias, yet, the questionnaires was anonymous allowing the participants to provide reliable answers.

## Conclusions

Students’ attitudes toward medically uninsured migrant patients declined as they progressed through school, with women having more favorable attitudes than men. Medical schools should attempt to foster the enthusiasm and idealism that students possess when they enter medical school in addition to providing clinical experiences with migrant patients and opportunities for cross- cultural communication [29].

## Supplementary information

**Additional file 1 Appendix 1.** MSATU modifications from English to the Israeli setting

**Additional file 2 Appendix 2**. Comparison between all 3839 medical students in medical schools in Israel in 2017 and the students who participated in the study

## Data Availability

Available by contacting the corresponding author. The MSATU use of the translated Hebrew version of the MSATU questionnaire was approved by Dr. Sonia J. S. Crandall.

## Abbreviation

MSATU: Medical Students’ Attitudes Toward the Underserved
