# Supplementary material for: Attitudes of Israeli medical students towards the medical treatment of uninsured migrants
Source: BMC Med Educ. 2020 Mar 14;20:72. doi: 10.1186/s12909-020-1973-4 (PMC7071779; doi:10.1186/s12909-020-1973-4)
Supplement: Supplementary file 1 — Additional file 1 Appendix 1. MSATU modifications from English to the Israeli setting [file 12909_2020_1973_MOESM1_ESM.docx]

Appendix 1: MSATU modifications from English to the Israeli setting:

| Characteristic | English version | Hebrew version |
| --- | --- | --- |
| Race/Ethnicity | White, African American, American Indian, Asian/Pacific Islander, Multicultural, Other, Don't know, Prefer not to answer | Jewish, Muslim, Christian, Druze, Other |
| Country of birth | Was not asked in the original survey | Any country |
| Are you Latino or Hispanic | Yes, no, don’t know, prefer not to answer | Not relevant |
| Name of Medical School | None | Any |
| Terminology | Medically needy or underserved | Migrant workers |
