# Supplementary material for: Attitudes of Israeli medical students towards the medical treatment of uninsured migrants
Source: BMC Med Educ. 2020 Mar 14;20:72. doi: 10.1186/s12909-020-1973-4 (PMC7071779; doi:10.1186/s12909-020-1973-4)
Supplement: Supplementary file 2 — Additional file 2 Appendix 2. Comparison between all 3839 medical students in medical schools in Israel in 2017 and the students who participated in the study [file 12909_2020_1973_MOESM2_ESM.docx]

Appendix 2: Comparison between all 3,839 medical students in medical schools in Israel in 2017 and the students who participated in the study

Table 1: Percentage of medical students in Israel, by gender in 2016

| Clinical | | Pre-clinical | | University |
| --- | --- | --- | --- | --- |
| Female | Male | Female | Male |  |
| 52.6 | 47.4 | 47.2 | 52.8 | 1 |
| 58.2 | 41.8 | 59.2 | 40.8 | 2 |
| 59.8 | 40.2 | 58.3 | 41.7 | 3 |
| 0.0 | 0.0 | 52.2 | 47.8 | 4 |
| 60.9 | 39.1 | 51.1 | 48.9 | 5 |

Table 2: Percentage of students responding to the MASTU questionnaire in 2016

| Clinical | | Pre-clinical | | Sample |
| --- | --- | --- | --- | --- |
| Female | Male | Female | Male |  |
| 58.9 | 41.1 | 37.8 | 62.2 | 1 |
| 61.1 | 38.9 | 48.9 | 51.1 | 2 |
| 54.1 | 45.9 | 53.5 | 46.5 | 3 |
| 0.0 | 0.0 | 46.9 | 53.1 | 4 |
| 60.5 | 39.5 | 50.4 | 49.6 | 5 |

Table 3: *P* values of *chi*-square tests evaluating the rates of students for each cell in tables 1 and 2

| Clinical | | Pre-clinical | | Sample |
| --- | --- | --- | --- | --- |
| Female | Male | Female | Male |  |
| 0.2 | 0.09 | 0.08 | 0.1 | 1 |
| 0.3 | 0.9 | 0.2 | 0.2 | 2 |
| 0.4 | 0.4 | 0.3 | 0.3 | 3 |
|  |  | 0.3 | 0.3 | 4 |
| 1 | 1 | 0.9 | 0.9 | 5 |
